# Magnitude of Anemia in Geriatric Population Visiting Outpatient Department at the University of Gondar Referral Hospital, Northwest Ethiopia: Implication for Community-Based Screening

**DOI:** 10.1155/2018/9869343

**Published:** 2018-01-08

**Authors:** Mulugeta Melku, Wondimu Asefa, Ahmed Mohamednur, Tesfahun Getachew, Bayechish Bazezew, Meseret Workineh, Bamlaku Enawgaw, Belete Biadgo, Zegeye Getaneh, Debasu Damtie, Betelihem Terefe

**Affiliations:** ^1^Department of Hematology and Immunohematology, School of Biomedical and Laboratory Sciences, College of Medicine and Health Sciences, University of Gondar, 6200 Gondar, Ethiopia; ^2^Department of Medical Laboratory Sciences, School of Biomedical and Laboratory Sciences, College of Medicine and Health Sciences, University of Gondar, Gondar, Ethiopia; ^3^Department of Immunology and Molecular Biology, School of Biomedical and Laboratory Sciences, College of Medicine and Health Sciences, University of Gondar, Gondar, Ethiopia; ^4^Department of Clinical Chemistry, School of Biomedical and Laboratory Sciences, College of Medicine and Health Sciences, University of Gondar, Gondar, Ethiopia

## Abstract

**Objective:**

This study is aimed at assessing the magnitude and its associated factors of anemia in geriatric population visiting outpatient department at the University of Gondar referral hospital, northwest Ethiopia.

**Method:**

A cross-sectional study was conducted among elder patients in Gondar town, North Gondar District, in May 2013. A total of 200 randomly selected geriatric population participated in the study. Summary statistics were computed and presented in tables and figure. Both bivariate and multivariable binary logistic regression were fitted to identify associated factors. A *P* value < 0.05 was considered as statistically significant.

**Result:**

The median age of the study participants was 65 years (Interquartile range (IQR): 8 years). The prevalence of anemia in the geriatric patients was 54.5% (*n* = 109), of which 61.5% (*n* = 67) were males. Mild type anemia was predominant, 55.96% (*n* = 61). Geriatric patients with an elevated erythrocyte sedimentation rate (AOR = 9.04, 95% CI: 4.2–19.7) and who are vegetarians (AOR = 2.2, 95% CI: 1.03–4.71) were at high risk of developing anemia.

**Conclusion:**

The magnitude of anemia was high in geriatrics. Mild anemia was the predominant type. Vegetarians and geriatrics with elevated erythrocyte sedimentation rate were more likely to develop anemia. Hence, early diagnosis and management of anemia have paramount importance to prevent adverse outcomes in geriatrics.

## 1. Background

Anemia is a decrease in the number of red blood cells or hemoglobin (Hb), resulting in a lower ability for the blood to carry oxygen to body tissues. As to World Health Organization (WHO) recommendation, anemia as Hb level < 12.0 g/dL for women and <13.0 g/L for men is most frequently used, even though the appropriateness in older populations may be questioned [1]. Anemia is a worldwide public health problem, with global prevalence estimated to be 24.8% (95% CI: 22.9–26.7). The majority of the global disease burden of anemia is shouldered by the developing world, with high prevalence in Africa and southeast Asia [2].

In geriatric age group, anemia is a common concern and public health problem [3–6]. It is frequently underdiagnosed and often not reported to the patient because it is mostly perceived as a mere consequence of aging or as a disease marker [7]. However, anemia has been implicated with severe complications. It can greatly hamper the quality of life and, consequently, it can have an impact on healthcare requirements and expenditure. Thus, it is becoming a significant healthcare burden [8, 9].

Evidences showed that anemia in elderly has been strongly linked with severe complications, including impaired physical functioning [10], decreased functionality [11], multidimensional loss of function [12], increased risk of frailty [13–15], depression [16], cognitive impairment [17], obstructive sleeping apnea [18], frequent comorbidity and hospitalization [19–21], and increased risk of death [20, 22, 23]. Despite old age being a major risk factor for anemia which threatens the quality of elderly life and has a substantial social and economic effects, anemia, however, should not be accepted as an inevitable consequence of aging, as it does reflect poor health and increased vulnerability to adverse outcomes in older persons [24, 25].

Anemia in elderly is multifactorial in etiology and a complex interaction of many factors. Causes of anemia in the elderly are divided into three groups: anemia of chronic disease, nutritional deficiency, and unexplained anemia [26]. These groups are not, however, mutually exclusive. In most of the cases, several of these causes may coexist and may each contribute independently to the anemia. The most common causes of anemia in the elderly are chronic diseases and iron deficiency [24]. Approximately, one-third of anemia in elderly person has attributed to nutrient deficiency; most of these cases are attributable to iron deficiency, including chronic blood loss. Moreover, folate deficiency and vitamin B12 deficiency are also causes of nutritional anemia and warrant routine screening. Although fortification of foodstuffs has made folate deficiency less common, more than 10% of elderly persons have borderline or low vitamin B12 levels [25, 27].

Although numerous studies on the prevalence of anemia in the elderly have been published, they vary markedly in study design and populations sampled and, consequently, in its prevalence range widely [3, 15, 21, 28, 29]. Despite the increasing size of the geriatric population in Ethiopia, much has not been done to determine the epidemiology of anemia in this group of the population.

## 2. Methods and Materials

### 2.1. Study Design, Period, Setting, Population, and Sampling Techniques

This institution based cross-sectional study was conducted in the outpatient department of University of Gondar Hospital in May 2013. The sample size was estimated by using single population proportion at 95% confidence interval, sampling error of 5%, and an estimated anemia prevalence of 50% in the elderly population. The sample size was corrected into 200, as the estimated number of elderly patients visiting the hospital during the study period was lower than 10,000. A systematic random sampling was applied; participants were selected at every second interval from the sequence of outpatient department visit.

On the basis of the proposed working definition of an “older person” in Africa for the Minimum Data Set (MDS) project [30], elderly patients with the age of 60 years and above for both men and women and who had not been transfused with red blood cells within the previous three months were included. Those elderly patients who were seriously ill and unable to respond to the questions during the time of data collection were excluded from the study for ethical reasons.

### 2.2. Data Collection Method

Sociodemographic and clinical history of the study participants were collected by using pretested structured questionnaire via interview and medical record review, respectively. Height and weight of each study participants were measured using standard scales as recommended by WHO. BMI was computed as weight (kg)/height (m^2^). Based on BMI, nutritional status was evaluated and grouped as follows: underweight (BMI ≤ 18.5), normal weight (BMI = 18.5–24.9), and overweight (BMI ≥ 25.0).

### 2.3. Laboratory Methods and Assessment of Anemia

Three milliliters of venous blood was drawn into K_3_EDTA tube and analyzed for complete blood count (CBC) and erythrocyte sedimentation rate (ESR). The CBC was determined using a Sysmex KX-21N (Sysmex Corporation Kobe, Japan) automated hematology analyzer. Anemia was defined according to the WHO criteria as an adjusted Hb concentration lower than 12.0 g/dL for women and 13.0 g/dL for men. Severity of anemia was graded as severe (Hb < 8.0 g/dL) and moderate (Hb: 8.0–10.9 g/dL) for both men and women and as mild (Hb: 11.0–12.9 g/dL for men and Hb: 11.0–11.9 g/dL for women) [1]. Morphological classification of anemia was done by using red cell indices, and the reference values used in this study were MCV (80–100 fl), MCHC (31–35%), and MCH (27–32 pg). We used IRIA (LiNEAR Chemicals, SL, Spain) for determination of ESR and a cutoff value of 20 mm/hr and 30 mm/hr was used for men and women, respectively.

### 2.4. Data Analysis and Interpretation

Data were entered to EPI info version 3.5.3 and then transferred to SPSS version 20 statistical package for analysis. Summary statistics were computed, and the results were presented in tables and figure. A binary logistic regression model was fitted to identify factors associated with anemia. Odds ratio, Chi-square, and 95% CI for odds ratio were computed to assess the strength of association and statistical significance in bivariate analysis. Variables having *P* less than or equal to 0.2 in bivariate binary logistic regression analysis were included in multivariable binary logistic regression analysis to control confounders. A *P* value less than 0.05 in the multivariable binary logistic regression model was considered to be statistically significant.

### 2.5. Ethical Consideration

The study was approved by an Institutional Review Board of the University of Gondar. The purpose and importance of the study were explained to each study participants. Written consent was obtained from each participant. To ensure confidentiality of participants' information, anonymous typing was used whereby the name of the participants and any participants' identifier were not written on the questionnaire. To keep the participants' privacy, they were interviewed alone in a separate room.

## 3. Results

### 3.1. Characteristics of Study Participants

Two hundred (*n* = 200) elderly patients participated in this study. The median age of the study participants was 65 years (IQR: 8 years). About one-third, (35%, *n* = 70), of them, belong to 60–64 years age group. Majority of the elderly patients were males (55%, *n* = 110), and most of the study participants reside in an urban setting (55.5%, *n* = 111). More than three-fourths (77%, *n* = 154), half (61%, *n* = 122), and one-third (43%, *n* = 86) of the participants were married, unable to read and write, and farmer by occupation, respectively. The average monthly family income of the study participants was 1093.36 Ethiopia Birr (ETB), of which 42% (*n* = 84) had an income of less than 1000 ETB. The proportion of elderly patients with a family size of greater than 6 accounts for 15.5% (*n* = 31) (Table 1).

### 3.2. Dietary Habit and ESR Value of Study Participants

About 78% (*n* = 156) and 69.5% (*n* = 139) of the study participants had a habit of consuming fruits less frequently and meat at least once a week, respectively. Moreover, 62% (*n* = 124) of them had a habit of taking coffee and/or tea after a meal, whereas 44.5% (*n* = 89) of them had a habit of consuming vegetables less frequently. Concerning the nutritional status and ESR status of the study participants, 26% (*n* = 52) and 70.5% (*n* = 141) of them were underweighted and had an elevated ESR value, respectively (Table 2).

### 3.3. Anemia Prevalence and Its Severity

**Figure 1:**
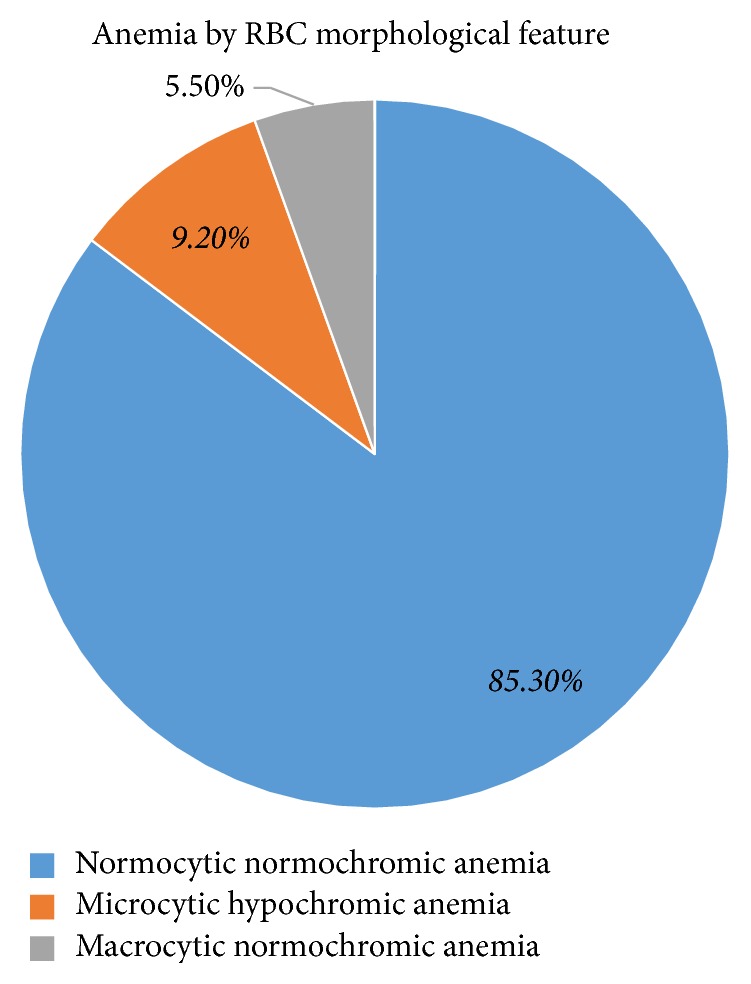
RBC morphological feature among anemic elderly study participants (*n* = 109).

The median (IQR) Hb concentration for males and females was 12.5 g/dl (IQR: 2.6 g/dl) and 12.15 g/dl (IQR: 2.85 g/dl), respectively. The prevalence of anemia in the elderly patients was 54.5% (95% CI: 47.54–61.46). Out of the total anemic elderly patients, 7 (6.4%), 40 (36.7%), and 62 (56.9%) had severe, moderate, and mild anemia, respectively (Table 3). Regarding morphologic feature of anemia, the majority of anemic patients had been suffering from normocytic normochromic anemia, 85.3% (*n* = 93) (Figure 1).

### 3.4. Factors Associated with Elderly Anemia

In bivariate binary logistic regression analysis, male sex, vegetarians, elevated ESR, underweight, and normal weight were significantly associated with elderly anemia. But in multivariable binary logistic regression analysis controlling the possible cofounders, only vegetarians and elevated ESR value were found to be factors statistically associated with elderly anemia. The odds of anemia in elderly patients who were vegetarians were two times (AOR = 2.2, 95% CI: 1.03–4.71) higher than the odds of anemia in elderly patients who had a habit of consuming meat at least once in every two weeks. Similarly, elderly patients who had an elevated ESR were nine times (AOR = 9.04, 95% CI: 4.2–19.7) more likely to be anemic as compared to those whose ESR value was within the normal range (Table 4).

## 4. Discussion

Anemia is a critical clinical problem in the elderly population, especially in hospitalized geriatric patients, and is known to be associated with increased morbidity and mortality. The present institutional based cross-sectional study found that the prevalence of anemia in elderly patients visiting outpatient department was high, and elevated ESR and being vegetarian were found to be strongly associated with geriatric anemia.

In this study, elderly patients had anemia prevalence of 54.5%, and according to WHO cutoff anemia has severe public health significance in this group [1]. It is comparable to the prevalence reported by Sahin et al. (54.9%) [31]. It is also comparable with a study reported by Tay and Ong (57.1%) [21], even though the study design and the criteria to define the population and anemia is different from us, as Tay and Ong study was retrospective in elderly hospitalized patients aged 65 and above year and the anemia was not defined according to WHO criterion. However, it is higher than the prevalence reported by Nakashima et al. (29%) [3], Sgnaolin et al. (12.8%) [32], and Bang et al. (8.33%) [10]. The possible reason for the discrepancies might be the characteristics differences between populations studied. In this study, the study participants were elderly patients who sought medical intervention in outpatients department, whereas in the case of Nakashima et al., Sgnaolin et al., and Bang et al. studies, the participants were institutionalized in long-term care [3] and community-dwelling elderly people [10, 32], who were assumed to be apparently healthy; thereby the risk of anemia is low. Similarly, the prevalence revealed in this study was higher than the prevalence (36.7%) reported from Reykjavik, Iceland [33], and the prevalence (10.6%) reported by NHANES III for black Americans [25]. This can be due to the differences in nutritional and environmental factors across the population studied.

On the other hand, the magnitude of anemia in this study was lower than studies carried out by Dunn et al. (77%) [34] and Kaur et al. (71%) [29]. This difference can be attributed to the distinctive characteristics of study participants, as Dunn et al. included elderly patients who were admitted to hospital for palliative care and Kaur et al. also included those admitted to in-patients in addition to outpatients; however, in our study only outpatients were considered. In elderly patients admitted to health facilities, the range and degree of comorbid conditions increase the likelihood of anemia development as compared with elderly outpatients. Contingent on these situations, the magnitude of anemia in the present study is lower than those studies conducted among elderly hospitalized patients.

Similar to previous studies carried out by different scholars [10, 21, 25, 35], this study found that the magnitude of anemia was higher in elderly men (60.9%) than women (46.7%). Moreover, a systematic review revealed that the estimates of anemia prevalence range from 2.9% to 61% in elderly men and from 3.3% to 41% in elderly women, but the threshold values are, in general, higher for men than for women [36]. The difference in the prevalence rates of anemia for men and women can be explained by the fact that in each decade beyond the age of 30, the concentration of free and bioavailable testosterone declines sharply in males. This negatively impacts the enhanced metabolic processes of the bone marrow. As testosterone level decreases with aging, the rate of erythropoiesis tends to be declined and predispose men to increased risk of anemia [37, 38]. In contrast to elderly men, the postmenopausal estrogen, which acts as an inhibitor of erythropoiesis, declines gradually as women age [39]. This intern would probably decrease the risk of anemia in elderly women as compared to men.

In this study, the odds of anemia in vegetarians were two times as high as the odds in those who had a habit of consuming meat at least once in every two weeks. Evidence suggested that the omission of meat and other animal products from the diet increases the risk of nutritional deficiencies [40, 41]. As a result, in vegetarians, the risk of vitamin B12, iron, and other several minerals deficiencies is common, which predispose them to an increased risk of nutritional deficiency anemia [42, 43]. Together with high magnitude of undernutrition which is a major public health concern in many African countries as a consequence of poverty and limitation of access to basic social supports [44] and aging related physiological changes, vegetarians are at higher risk of developing anemia.

In this study, we noticed that elderly patients who had an elevated ESR were nine times (AOR = 9.04, 95% CI: 4.2–19.7) more likely to be anemic as compared to those whose ESR value was within the normal range. The systemic inflammation associated with aging as well as chronic/acute conditions has been inferred from commonly used sensitive laboratory tests like ESR and C-reactive protein [45]. It is known that elevated ESR value is strongly correlated with the inflammatory response. Inflammatory conditions result in the accumulation of reactive oxygen species which activate nuclear factor kappa B, a transcription factor playing a central role in activating the innate immune response and inducing the expression of numerous proinflammatory cytokines. These pro-inflammatory cytokines further induce the expression of hepcidin antimicrobial peptide, a critical mediator of anemia of inflammation, which decreases serum iron availability and correlates with serum ferritin [46]. Moreover, patients with inflammatory diseases have been demonstrated to have decreased red cell survival, disorders of erythropoiesis, low erythropoietin response to hypoxia, and progressive erythroid progenitor cell resistance to erythropoietin stimulation [47]. Thus, elderly people having elevated ESR value are at higher risk of developing anemia.

## 5. Limitation of the Study

One of the limitations of this study is that only hemoglobin measurement was used to define anemia; the micronutrient analysis was not done for the assessment of nutritional deficiency anemia. The second limitation is that only ESR was used as an indicator of inflammation; other sensitive indicators like serum C-reactive protein, albumin, and cytokines were not measured. The third limitation of this study is related to the study design and the sample size, as its cross-sectional nature with small sample size.

## 6. Conclusion

In conclusion, the prevalence of anemia was found to be high, indicating that it is a severe public health problem in the study area. Being vegetarians and elevated ESR value have been strongly associated with high prevalence of anemia in elderly outpatients. Hence, provision of community-based anemia screening and nutritional educations are advisable to improve the quality of life and reduce the complications in the elderly population. Moreover, further community-based studies, employing longitudinal design and preferably involving biochemical analysis, are required to identify risk factors as well as to estimate the relative contribution of nutritional status to anemia in this group of the population.

## Figures and Tables

**Table 1:** Sociodemographic characteristics of study participants (*n* = 200).

Variables	Frequency	Percent
*Age (years)*		
60–64	70	35
65–69	54	27
70–74	38	19
>74	38	19
*Sex*		
Female	90	45
Male	110	55
*Residence*		
Urban	111	55.5
Rural	89	44.5
*Marital status*		
Single	1	0.5
Married	154	77
Divorced	15	7.5
Widowed	30	15
*Educational level*		
Unable read and write	122	61
Attended primary school	32	16
Attended secondary school	27	13.5
Attended higher education	19	9.5
*Occupation*		
Daily laborer	9	4.5
Private employee	21	10.5
Governmental employee	37	18.5
Farmer	86	43
Housewife and retired	47	23.5
*Family size*		
1–3 members	54	27
4–6 members	115	57.5
>6 members	31	15.5
*Monthly income (ETB)*		
<1000	84	42
100–1500	90	45
>1500	26	13

**Table 2:** Dietary habit, nutritional status, and ESR value of study participants (*n* = 200).

Variables	Frequency	Percentage
*Meat consumption habit*		
Consumed at least once a week and more	139	69.5
Vegetarians	61	30.5
*Frequency of fruit consumption*		
At least once a week and more	44	22
Less frequently	156	78
*Frequency of vegetable consumption*		
At least once a week and more	111	55.5
Less frequently	89	44.5
*Coffee or tea consumption habit after meal*		
Yes	124	62
No	76	38
*Nutritional status, BMI*		
Underweight	52	26
Normal	133	66.5
Overweight	15	7.5
*ESR*		
Normal	59	29.5
Elevated	141	70.5

BMI: Body mass index; ESR: erythrocyte sedimentation rate.

**Table 3:** Summary of anemia severity among anemic study participants (*n* = 109).

Variables	Frequency	Percentage
*Anemia by severity*		
Mild	62	56.9
Moderate	40	36.7
Severe	7	6.4
Total	109	54.5

**Table 4:** Analysis of factors associated with anemia among study participants (*n* = 200).

Variables	Anemia		COR (95% CI)	AOR (95% CI)
	Anemic (%)	Non-anemic (%)		
*Sex*				
Male	67 (60.9)	43 (39.1)	1.78 (1.01, 3.13)^b^	1.53 (0.79, 2.96)
Female	42 (46.7)	48 (53.3)	1.00	
*Residence*				
Urban	56 (50.5)	45 (49.5)	1.00	
Rural	53 (59.6)	36 (40.4)	1.45 (0.82, 2.54)	
*Age group (year)*				
60–64	33 (47.1)	37 (52.9)	1.00	
65–69	35 (64.8)	19 (35.2)	2.10 (0.99, 4.30)^+^	
70–74	19 (50)	19 (50)	1.12 (0.51, 2.47)	
>74	22 (57.9)	16 (42.1)	1.54 (0.70, 3.42)	
*Marital Status*				
Married	80 (51.9)	74 (48.1)	1.00	
Single or divorced	10 (62.5)	6 (37.5)	1.54 (0.53, 4.45)	
Widowed	19 (63.3)	11 (36.7)	1.60 (0.71, 3.58)	
*Educational status*				
Unable read and write	68 (55.7)	54 (44.3)	1.14 (0.64, 2.01)	
Attended primary school or above	41 (52.6)	37 (47.4)	1.00	
*Occupation*				
Daily laborers, housewives and retired	28 (50)	28 (50)	0.76 (0.36, 1.58)	
Farmers	48 (55.8)	38 (44.2)	0.9 (0.5, 1.87)	
Employed (private and government)	33 (56.9)	25 (43.1)	1.00	
*Family size*				
1–3 members	26 (48.1)	28 (51.9)	1.00	
4–6 members	64 (55.7)	51 (44.3)	1.35 (0.71, 2.58)	
>6 members	19 (61.3)	12 (38.7)	1.71 (0.69, 4.2)	
*Meat consumption habit*				
Consumed at least once every two weeks and more	69 (49.6)	70 (50.4)	1.00	
Vegetarians	40 (65.6)	21 (34.4)	1.93 (1.04, 3.61)	2.2 (1.03, 4.71)^*∗*^
*Frequency of Fruit consumption*				
At least once a week and more	23 (52.3)	21 (47.7)	1.00	
Less frequently	86 (55.1)	70 (44.9)	1.12 (0.57, 2.2)	
*Frequency of Vegetable consumption*				
At least once a week and more	61 (55)	50 (45)	1.00	
Less frequently	48 (53.9)	41 (46.1)	0.96 (0.55, 1.68)	
*Coffee or tea consumption habit after meal*				
Yes	65 (52.4)	59 (47.6)	0.8 (0.45, 1.43)	
No	44 (57.9)	32 (42.1)	1.00	
*Monthly income (ETB)*				
<1000	45 (53.6)	39 (46.4)	0.99 (0.41, 2.39)	
100–1500	50 (55.6)	40 (44.4)	1.07 (0.45, 2.57)	
>1500	14 (53.8)	12 (46.2)	1.00	
*ESR*				
Normal	12 (20.3)	47 (79.7)	1.00	
Elevated	97 (68.8)	44 (31.2)	8.63 (4.17, 17.87)	9.04 (4.2, 19.7)^*∗*^
*Nutritional status, BMI*				
Underweight	33 (63.5)	19 (36.5)	6.95 (1.74, 27.76)^b^	4.02 (0.81, 19.93)
Normal	73 (54.9)	60 (45.1)	4.87 (1.31, 18.1)^b^	3.61 (0.8, 16.25)
Overweight	3 (20%)	12 (32)	1.00	

ETB: Ethiopian Birr; BMI: body mass index; COR: crude odds ratio; AOR: adjusted odds ratio; and CI: confidence interval. ^+^Category of a variable with *P* value less than 0.2 in bivariate binary logistic regression analysis, ^b^Category of a variables which were statistically significant in bivariate binary logistic regress but not in multivariable analysis. ^*∗*^Category of a variables which were statistically significant in multivariable binary logistic regression analysis (*P* value < 0.05).

## Abbreviations

AOR: Adjusted odds ratio
BMI: Body mass index
CBC: Complete blood count
CI: Confidence interval
COR: Crude odds ratio
ESR: Erythrocyte sedimentation rate
ETB: Ethiopian Birr
IQR: Interquartile range
Hb: Hemoglobin
MCH: Mean cell hemoglobin
MCHC: Mean cell hemoglobin concentration
MCV: Mean cell volume
MDS: Minimum Data Set
SD: Standard deviation
WHO: World Health Organization.

## Data Access

Data will be available upon request from the corresponding author.
